# Enhancing solubility of deoxyxylulose phosphate pathway enzymes for microbial isoprenoid production

**DOI:** 10.1186/1475-2859-11-148

**Published:** 2012-11-14

**Authors:** Kang Zhou, Ruiyang Zou, Gregory Stephanopoulos, Heng-Phon Too

**Affiliations:** 1 Chemical and Pharmaceutical Engineering, Singapore-MIT Alliance, 4 Engineering Drive 3, Singapore, Singapore; 2 Department of Biochemistry Yong Loo Lin School of Medicine, National University of Singapore, 8 Medical Drive Blk MD7 #05-04, Singapore 117597, Singapore; 3 Department of Chemical Engineering, Massachusetts Institute of Technology, 77 Massachusetts Avenue, Cambridge, USA

**Keywords:** Isoprenoids, Protein solubility, Deoxyxylulose phosphate pathway, Activity analysis, Metabolic engineering

## Abstract

**Background:**

Recombinant proteins are routinely overexpressed in metabolic engineering. It is well known that some over-expressed heterologous recombinant enzymes are insoluble with little or no enzymatic activity. This study examined the solubility of over-expressed homologous enzymes of the deoxyxylulose phosphate pathway (DXP) and the impact of inclusion body formation on metabolic engineering of microbes.

**Results:**

Four enzymes of this pathway (DXS, ISPG, ISPH and ISPA), but not all, were highly insoluble, regardless of the expression systems used. Insoluble dxs (the committed enzyme of DXP pathway) was found to be inactive. Expressions of fusion tags did not significantly improve the solubility of dxs. However, hypertonic media containing sorbitol, an osmolyte, successfully doubled the solubility of dxs, with the concomitant improvement in microbial production of the metabolite, DXP. Similarly, sorbitol significantly improved the production of soluble and functional ERG12, the committed enzyme in the mevalonate pathway.

**Conclusion:**

This study demonstrated the unanticipated findings that some over-expressed homologous enzymes of the DXP pathway were highly insoluble, forming inclusion bodies, which affected metabolite formation. Sorbitol was found to increase both the solubility and function of some of these over-expressed enzymes, a strategy to increase the production of secondary metabolites.

## Introduction

Isoprenoids, a large family of natural compounds including many plant based pharmaceuticals such as artemisinin [1] and paclitaxel [2], are produced by the deoxyxylulose phosphate (DXP) pathway and/or the mevalonate (MVA) pathway in nature [3]. The current industrial isoprenoid production methods include direct extraction from plants and semi-synthesis using plant metabolites [4]. These processes are all restricted by the supply of specific plant materials, which are often affected by unpredictable factors including variations in weather and market fluctuations [5]. To improve sustainability and production capacity of isoprenoids, heterologous biosynthesis from economical carbon sources in microbes has been intensively studied in the past decade [2,5]. To date, there have been many successful reports of using the DXP pathway to produce isoprenoid nutraceuticals (e.g. lycopene [6-10] and other carotenoids [11,12]) and pharmaceuticals (e.g. artemisinin precursors [13,14] and paclitaxel precursors [2]).

To increase carbon flux through the DXP pathway, the enzymes involved were overexpressed [2,6-14] to catalyze the bio-transformations of the DXP metabolites *in vivo*. So far, four enzymes (DXS, IDI, ISPD and ISPF) have been identified to be rate limiting based on a series of empirical studies [9,15]. The expression levels of these four enzymes have also been semi-empirically optimized for paclitaxel precursor production [2]. It is well accepted that some recombinant proteins can form insoluble aggregates (termed as inclusion bodies), generally regarded to be functionally inactive [16]. However, the extents to which the over-expressed recombinant endogenous DXP enzymes forming inclusion bodies and their impacts on the flux through the pathway have yet to be systematically investigated.

This study examined the solubility status of all the DXP enzymes when overexpressed and attempted to demonstrate the importance of protein solubility in the production of secondary metabolites. Computational prediction was initially explored to evaluate the solubility status and empirical verifications were carried out in *E. coli*. An unanticipated and critical observation is that many DXP enzymes (DXS, ISPA, ISPG and ISPH) were found to be highly insoluble. Interestingly, the enzymes IDI, ISPD and ISPF, thought to be rate-limiting and hence useful for the enhancement of isoprenoids production [9], were found to be highly soluble. From these observations, it is now necessary to reevaluate the use of the other highly insoluble DXP enzymes for enhancing isoprenoid production. Attempts were also made to optimize the solubility of the insoluble enzymes and to examine the enhancements in isoprenoid production.

## Results

### Solubility of over-expressed recombinant DXP pathway enzymes

**Figure 1:**
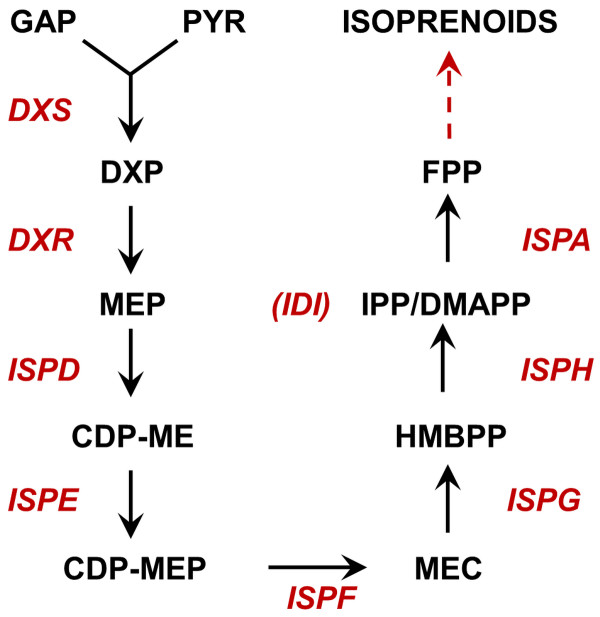
**Metabolites and enzymes related to the DXP pathway.** GAP: glyceraldehyde-3-phosphate; PYR: pyruvate; DXP: 1-deoxy-D-xylulose 5-phosphate; MEP: 2C-methyl-D-erythritol 4-phosphate; CDP-ME: 4-diphosphocytidyl-2C-methyl D-erythritol; CDP-MEP: 4-diphosphocytidyl-2C-methyl D-erythritol 2-phosphate; MEC: 2C-methyl-D-erythritol 2,4-diphosphate; HMBPP: (E)-4-hydroxy-3-methyl-but-2-enyl pyrophosphate; IPP: Isopentenyl diphosphate; DMAPP: Dimethylallyl diphosphate; FPP: Farnesyl diphosphate; DXS: 1-deoxy-D-xylulose 5-phosphate synthase; DXR: 1-deoxy-D-xylulose 5-phosphate reductase; ISPD: 4-diphosphocytidyl-2C-methyl-D-erythritol synthase; ISPE: 4-diphosphocytidyl-2-C-methylerythritol kinase; ISPF: 2-C-methyl-D-erythritol 2,4-cyclodiphosphate synthase; ISPG: 1-hydroxy-2-methyl-2-(E)-butenyl 4-diphosphate synthase; ISPH: 1-hydroxy-2-methyl-2-(E)-butenyl 4-diphosphate reductase; IDI: isopentenyl diphosphate isomerase; ispA: farnesyl diphosphate synthase.

DXP pathway has so far been characterized to be a linear pathway [3], producing isopentenyl diphosphate (IPP) and dimethylallyl diphosphate (DMAPP) from pyruvate and glyceraldehyde 3-phosphate (GAP), two important metabolites in central metabolism (Figure 1). IPP and DMAPP (C5) are further assembled into geranyl diphosphate (GPP, C10) and farnesyl diphosphate (FPP, C15), precursors for all C10 and C15 isoprenoids [4]. To date, little is known of the solubility of the enzymes involved in this pathway when overexpressed for the production of isoprenoids.

As a first attempt, the solubility of the enzymes in the DXP pathway was evaluated by *in silico* modeling. Several correlation algorithms built on published experimental data have been reported [17-21]. Revised WH method [18], one of the most commonly used and accurate methods [21], was used to predict solubility of the DXP pathway enzymes. Some of the enzymes (DXS, ISPE and ISPG) were predicted by these methods to be insoluble when overexpressed in *E. coli* (Table 1). Similarly, *in vitro* expression study [22] showed that a subgroup of the DXP pathway proteins (DXS and IDI) were insoluble (Table 1). As *in silico* prediction did not completely agree with the published *in vitro* expression data, it was essential to determine the solubility of the enzymes when overexpressed *in vivo*.

**Table 1:** **Solubility of the enzymes in the DXP pathway predicted *in silico *and determined *in vitro***

**Protein**	***In silico *predicted solubility **[18]	***In vitro *determined solubility **[22]
DXS	insoluble	22%
DXR	soluble	N.D.*
ISPD	soluble	67%
ISPE	insoluble	100%
ISPF	soluble	84%
ISPG	insoluble	N.D.
ISPH	soluble	75%
IDI	soluble	32%
ISPA	soluble	85%

*N.D.: not detected.

**Figure 2:**
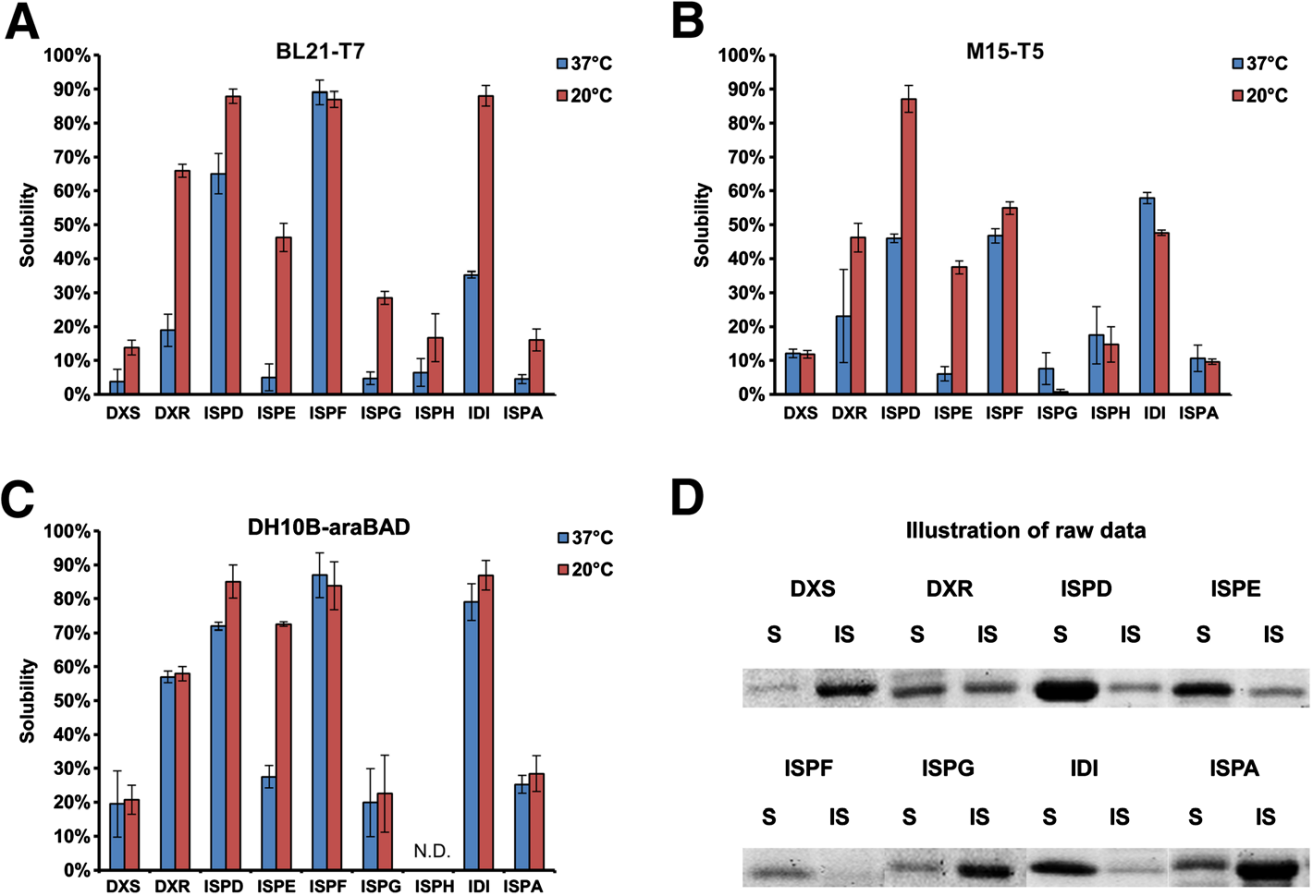
**Solubility of DXP enzymes in *E. coli.*** The expression systems and incubation temperatures played important roles in production of soluble proteins. All the enzymes in the DXP pathway were individually expressed by three expression systems (**A**: BL21 strain - T7 promoter; **B**: M15 strain - T5 promoter; **C**: DH10B strain - araBAD promoter) at two temperatures (37°C and 20°C). Presented data were average of triplicates and standard errors were drawn on the plot. The quantifications were based on image of SDS-PAGE gels, some of which (the set for DH10B strain – araBAD promoter, 20°C) were demonstrated in **D**. S: soluble protein fraction; IS: insoluble protein fraction. The pictures of DXP proteins were cropped from individual gel images and aligned for demonstration purpose. The protein solubilities were generally higher in 20°C than those in 37°C in all systems (Figure 2A-C).

To verify that some of the DXP enzymes when overexpressed were differentially soluble, each of the enzymes was expressed individually in three distinct expression systems in different strains of *E. coli* (BL21 strain - T7 promoter, M15 strain - T5 promoter and DH10B strain – araBAD promoter) at two temperatures (37°C and 20°C). The standard dosage of inducers were used to trigger expression of the proteins (10mM L-arabinose or 1mM IPTG [6,23]). In general, solubility of the proteins varied significantly (5% to 90%, Figure 2). The large variances in solubility across proteins suggested that the method for identifying and quantifying protein solubility is unbiased. This protein solubility analysis method (similar to that used in [21]) was also further validated by filtration studies (Additional file 1). A group of the DXP enzymes (DXS, ISPG, ISPH and ISPA) were indeed found to be highly insoluble (solubility less than 30% in all conditions examined) (Figure 2). DXS (the committed enzyme of DXP pathway) was previously identified to be crucial (rate-limiting) for isoprenoid production, was found to be highly insoluble in the present study. Using dxs as a prototype of highly insoluble enzymes, we next examined the impact of inclusion body formation on metabolic engineering of *E. coli* for isoprenoid production.

### Enzymatic activity of insoluble recombinant DXS

**Figure 3:**
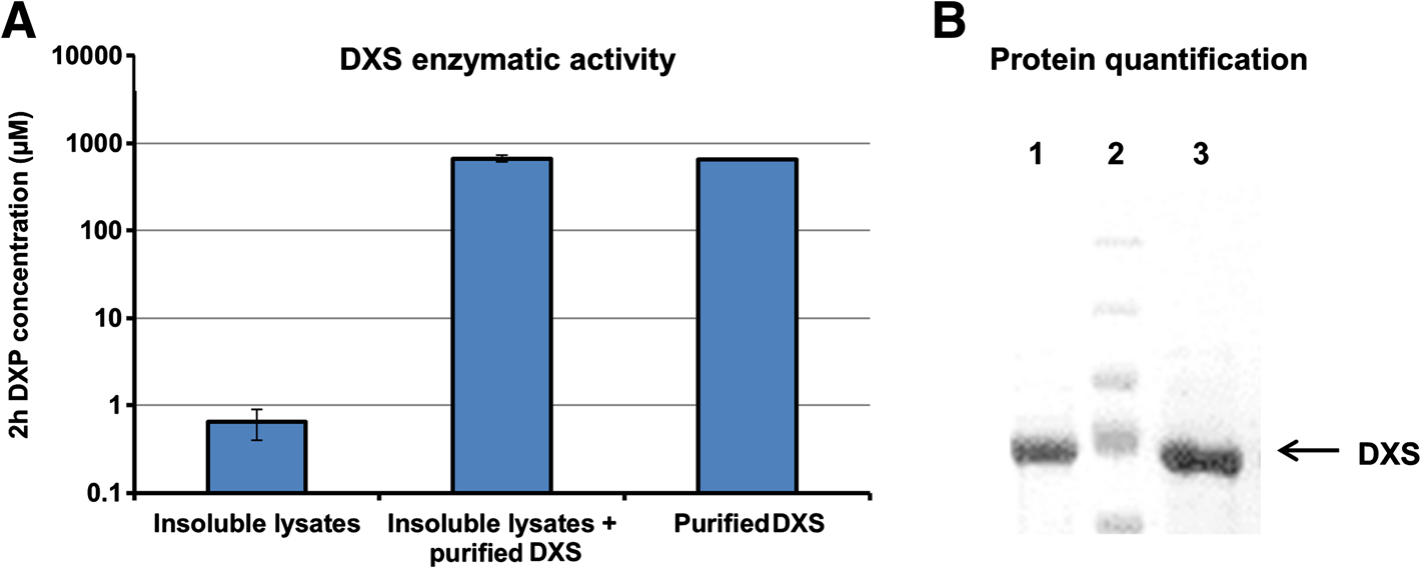
***In vitro *activity analysis of insoluble DXS.** Activity of insoluble DXS was found to be much lower than that of equal amount of soluble DXS by quantifying the quantity of DXP they produced *in vitro*. **A**: Quantification of the *in vitro* produced DXP by insoluble DXS, insoluble DXS + purified DXS (soluble) and purified DXS (soluble), presented data were average of triplicates and standard errors were drawn on the plot; **B**: Quantification of the insoluble DXS and the purified DXS (soluble) used in A, lane 1: the cell lysate containing insoluble DXS, lane 2: protein markers, lane 3: purified soluble DXS, the arrowed band was DXS.

Although some inclusion bodies formed with certain enzymes were reported to be active [24], it is generally accepted that inclusion bodies contain primarily incorrect folded proteins and are functionally inactive [25]. To test whether insoluble DXS is catalytically functional, lysates containing recombinant insoluble DXS was characterized by an *in vitro* assay, where DXS activity was determined by measuring the formation of DXP. It was found that DXP was produced at low levels (less than 1 μM) with insoluble DXS containing lysates. As a comparison, the same amount of purified soluble DXS (Figure 3 B) was spiked into the lysates, and high level of DXP (~700 μM) was produced (Figure 3 A), confirming that specific activity of insoluble DXS was significantly lower than that of soluble DXS. This observation suggested that strategies to increase the solubility of DXS may confer higher activity and metabolic flux for isoprenoid production *in vivo*.

### Improving solubility of DXS enhanced the production of DXP

**Figure 4:**
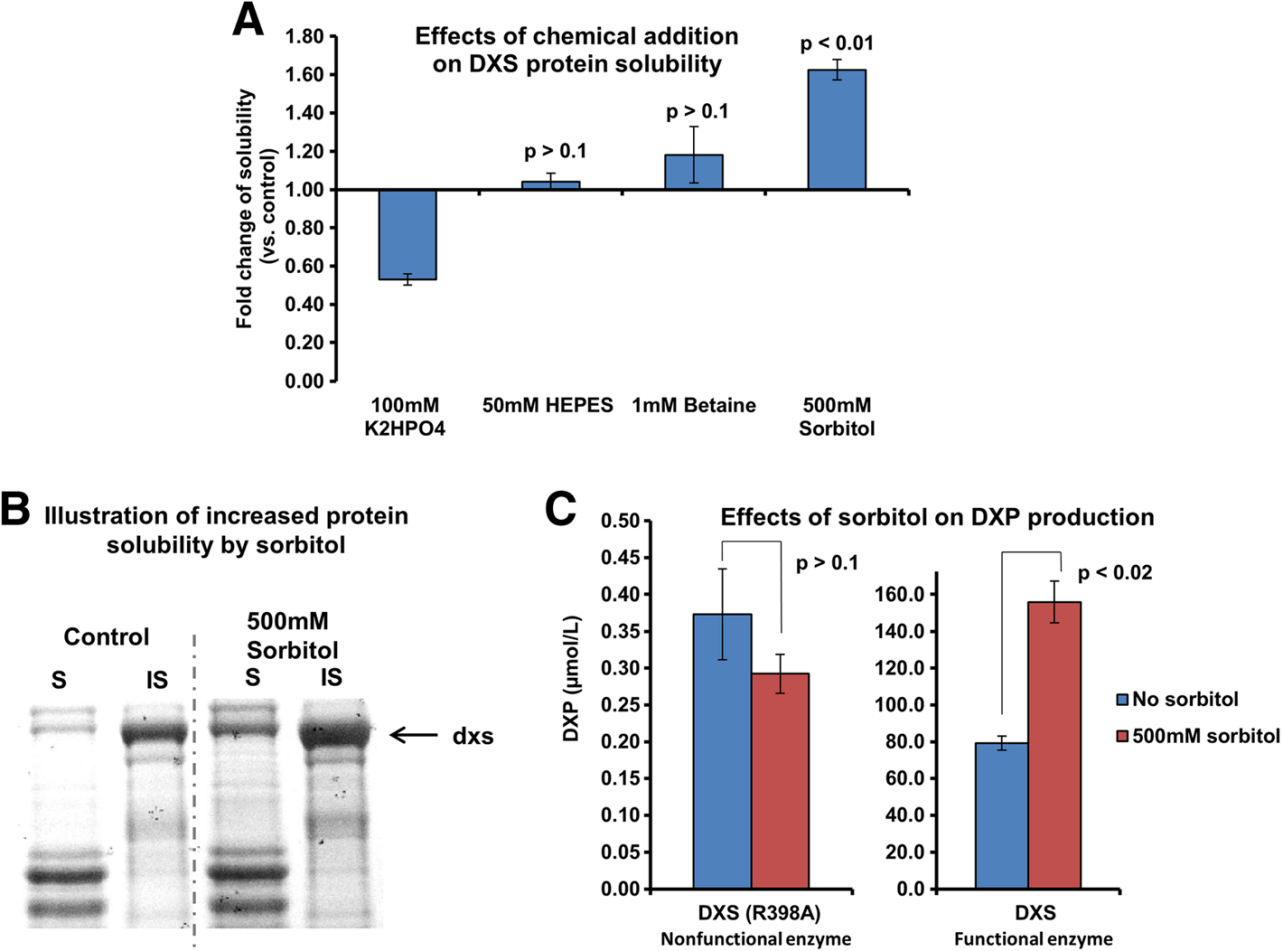
**Addition of chemicals for increasing solubility of DXS and production of deoxyxylulose phosphate (DXP).** Chemicals with different properties were used to increase solubility of dxs. The expression system (araBAD promoter-DH10B strain) and the temperature (20°C) which produced the most soluble DXS were used here. The use of high concentration of sorbitol was found to significantly increase solubility of DXS and production of DXP (product of DXS). **A**: Effects of the chemical additions on solubility of DXS; **B**: Illustration of increased protein solubility by sorbitol, S: soluble fraction, IS: insoluble fraction; the arrowed band was DXS; **C**: Effects of sorbitol addition on production of DXP; addition of sorbitol increased DXP production in the cells expressing DXS but not in the ones expressing DXS (R398A), a nonfunctional mutant. Presented data were average of triplicates and standard errors were drawn on the plot. Student’s t-test was used to calculate the p values in the statistical analysis.

Improvement of recombinant protein solubility has been intensively studied for the purpose of overproducing soluble proteins, and various effective strategies have been reported, such as lowering incubation temperature [25], use of fusion partner [26], overexpression of chaperone proteins [27] and protein mutagenesis [28]. Recently, Prasad et al. reported a simple yet effective approach to increase the solubility of recombinant proteins, where sorbitol at high concentration reduced protein aggregation in *E. coli*[29]. To test if this approach could increase the solubility of DXS, high concentration of sorbitol was added directly to the cells in culture. The solubility of DXS was examined and found to be significantly increased (Figure 4 A and B). Other chemicals, including osmolytes (betaine [30]) and buffering agents (HEPES, phosphate) did not improve the solubility of dxs significantly (Figure 4 A). In addition, sorbitol similarly improved the solubility of some but not all other DXP enzymes (Additional file 2).

To demonstrate that improved solubility of DXS results in enhanced production of DXP (committed metabolic intermediate in the DXP pathway), cells grown in sorbitol were lysed and the extracts quantified by LC-MS. It was found that concentrations of DXP were significantly higher in sorbitol treated cells as compared to control cells (Figure 4 C). Addition of sorbitol did not alter the production of DXP with cells overexpressing a nonfunctional DXS (Figure 4 C, the construction and characterization of the nonfunctional dxs was described in Additional file 3), indicative that the effect of sorbitol on cells overexpressing functional enzyme was likely be due to the increase in the concentration of soluble DXS. A parallel increase in the concentrations of MEP and MEC (Figure 1) were also observed (Additional file 4), suggesting that sorbitol increased the flux through the entire DXP pathway in cells overexpressing DXS.

### Improvement of ERG12 solubility and overproduction of mevalonate phosphate

**Figure 5:**
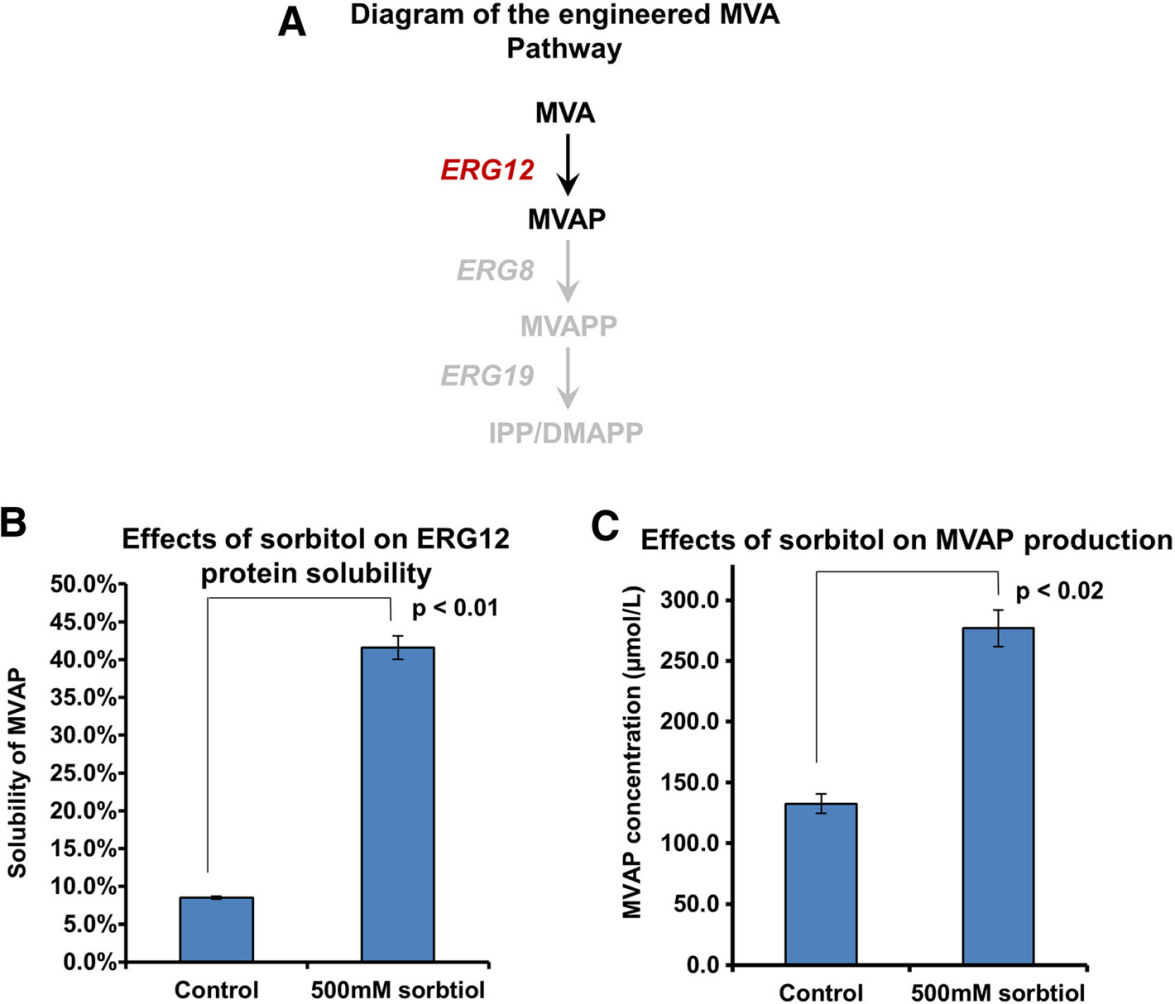
**Addition of chemicals for increasing solubility of ERG12 and production of mevalonate phosphate (MVAP).** The use of high concentration of sorbitol was also found to increase solubility of ERG12, a critical enzyme in the mevalonate pathway (the other isoprenoid precursor production pathway), and production of MVAP (product of ERG12). **A**: Metabolites and enzymes related to the mevalonate (MVA) pathway; MVA: Mevalonate, MVAP: Mevalonate phosphate, MVAPP: Mevalonate diphosphate, IPP: Isopentenyl diphosphate, DMAPP: Dimethylallyl diphosphate; ERG12: mevalonate kinase, ERG8: mevalonate phosphate kinase, ERG19: mevalonate diphosphate decarboxylase; **B**: Effects of sorbitol addition on solubility of ERG12, S: soluble fraction, IS: insoluble fraction; **C**: Effects of sorbitol addition on production of MVAP; Since ERG8 and ERG19 were not present in *E. coli* and also not recombinantly expressed, MVAP accumulated in ERG12 expressing *E. coli* , directly indicating activity of ERG12. Presented data were average of triplicates and standard errors were drawn on the plot. Student’s t-test was used to calculate the p values in the statistical analysis.

To extend the observation of the effect of sorbitol, a critical enzyme (ERG12) in the mevalonate pathway (the other isoprenoid precursor producing pathway, Figure 5 A) was investigated. More than half of overexpressed ERG12 was insoluble and sorbitol was similarly found to enhance the solubility of this enzyme (Figure 5 B). In line with the hypothesis that increased solubility confers higher enzymatic activity and better productivity of the respective metabolite, the production of mevalonate phosphate (MVAP) was doubled in the presence of high concentrations of sorbitol (Figure 5 C). Since the MVA pathway is not endogenous to *E. coli* (Figure 5 A), the production and accumulation of MVAP was attributed to the enzymatic activity of ERG12.

## Discussion

This study addressed an important and often overlooked issue of the solubility of over-expressed recombinant homologous or heterologous enzymes in metabolic engineering. Specifically, we investigated the solubility status of overexpressed DXP enzymes and a heterologous enzyme of the MVA pathway and the impacts on the production of critical precursor metabolites (DXP or MVAP), which are building blocks of all the isoprenoids. It was unexpected that four out of nine enzymes in DXP pathway (DXS, ISPA, ISPG and ISPH) were highly insoluble, despite being endogenous enzymes. Overexpression of DXS resulted in the accumulation of highly insoluble and non-functional (< 1% activity of the equivalent soluble form) enzyme. This observation cautions against the assumption that overexpression of an enzyme necessarily confers higher enzymatic activity. Interestingly, the combinatorial screening study [9] based on this contentious assumption identified three rate-limiting DXP enzymes (IDI, ISPD and ISPF), which incidentally were found to be highly soluble (Figure 2). It is thus not unreasonable to speculate that the previously thought to be ‘non rate-limiting’ enzymes found to be insoluble in this study, may serve to enhance the productions of isoprenoids when expressed in soluble forms.

Using DXS as a model enzyme, four commonly used fusion partners, trxA [31], nusA [18], slyD [32] and malE [33] were fused at the N-terminus of DXS in the attempt to increase solubility. The use of these fusion partners did not significantly increase the solubility of DXS (Additional file 5). The effectiveness of the fusion partners in enhancing protein solubility is largely protein-dependent and unpredictable [26,34]. Cysteine residues on surface of DXS (C32, C330 and C457), may form non-specific disulfide bonds and result in protein aggregation [28]. Site-directed mutagenesis of these residues also did not improve solubility (Additional file 6), suggesting that the aggregation of dxs protein was not due to disulfide bond mediated interactions.

Osmolytes have been shown to improve overexpressed proteins in *E. coli*[29]. Sorbitol at high concentrations significantly improved DXS solubility and the production of the metabolic product (DXP) in *E. coli*, indicating that solubility of recombinant enzymes is an important factor in the production of secondary metabolites. Consistent with this suggestion was that ERG12, another model enzyme, also showed improved solubility and secondary metabolite production in the presence of sorbitol. The reason why the metabolic intermediates (DXP, MEP and MEC etc.) instead of final product (lycopene etc.) were used as read-out for characterization of DXS was that rate limiting step (ISPG) existed between the intermediates and the isoprenoid products (Zhou et al. PLoS One, In Press, Additional file 7). It is worthy to note that the use of sorbitol is acceptable as a proof-of-concept but may not be routinely used in microbial fermentation due simply to the cost involved [29]. An alternative is to modify the host microbes (such as manipulation of cellular protein folding system [27,35]) to render these proteins more soluble for industrial applications.

## Conclusion

In this study, about half of the nine DXP proteins (DXS, ISPG, ISPH and ISPA) were found to be highly insoluble when overexpressed in *E. coli*. Insoluble DXS, the committed enzyme of the DXP pathway, showed significantly less enzymatic activity when compared to the equivalent amount of soluble enzyme *in vitro*. High concentration of sorbitol successfully increased the solubility of DXS and resulted in a parallel increase in the metabolic product (DXP). The strategy also improved both solubility and secondary metabolite production of ERG12, a critical enzyme in the mevalonate pathway. This study highlighted the importance of protein solubility in metabolic engineering of microbes for the overproduction of isoprenoids.

## Methods

### Bacteria strains and plasmids

Bacteria strains and plasmids used in this study were summarized in Additional file 8. All the DXP genes were amplified from *E. coli* genomic DNA and cloned into the modified pBAD-B (Invitrogen), pET-11a (Stratagene) and pQE30 (Qiagen) plasmids with 6xhis tag, SacI, XhoI restriction enzyme sites. Fusion partners (trxA, nusA, malE and slyD) were amplified from *E. coli* genomic DNA and cloned into pBAD-dxs with NcoI and SacI sites. Erg12 was amplified from *S. cerevisiae* genomic DNA and cloned into the modified pBAD-B plasmid with 6xhis tag, SacI and XhoI restriction enzyme sites. Dxs mutants R398A [36], C32A, C330A, C457A and C32A-C330A-C457A were generated according the ‘megaprimer’ protocol [37]. Primers used in this study were summarized in Additional file 8. All the pET-11a, pBAD-B and pQE30 based plasmids were transformed into *E. coli* BL21-Gold (DE3), *E. coli* DH10B and *E. coli* M15 respectively. pAC-LYC was co-transformed with all the plasmids except pBAD-erg12.

### *E. coli* growth and induction of protein expression

A colony was picked from agar plate, inoculated into 2xPY medium (20g/L Peptone, 10g/L Yeast extract, and 10g/L NaCl, pH=7) containing proper antibiotics, and incubated overnight. Ten microliter aliquots of overnight grown cell culture were inoculated into 1mL 2xPY medium in 14mL Falcon tube. Cells were grown at 37°C/300rpm till OD595 reached the range of 0.5~1.0. The cells were then induced with 1mM IPTG (*E. coli* BL21-Gold (DE3) and *E. coli* M15) or 10mM L-arabinose (*E. coli* DH10B) and grown at 37°C or 20°C for indicated time before collected for protein solubility assay or metabolite assay. Additives (sorbitol, betaine, phosphate, HEPES, mevalonate etc.) were also fed to cell culture upon induction if necessary.

### Prediction and quantification of protein solubility

The revised WH algorithm [18] was used for prediction of protein solubility. Protein solubility was experimentally quantified by centrifugation [21] as described below. At 24h after induction, cell suspension equivalent to 1mL OD595=1.0 cells, was centrifuged, and the pellet was resuspended in 100uL B-PERII reagent (Pierce). The mixtures were vortexed at room temperature for 10min, and centrifuged at 16,000g for 10min. The supernatant containing soluble cell lysates, and the pellets (resuspended in 100uL 2% w/v SDS) containing insoluble cell lysates were analyzed by SDS-PAGE. The SDS-PAGE gel was visualized by staining with instant blue (Gentaur), and image of the gel was processed and quantified by the software Quantity One (Bio-Rad). Protein solubility was defined as the quantity of the target protein in soluble cell lysates over that in total cell lysates (soluble cell lysates + insoluble cell lysates). Because ERG12 protein cannot be separated from an abundant endogenous protein on SDS-PAGE, it was detected by western blot analysis with anti-6xhis tag antibody (Penta-his Ab, Qiagen).

### *In vitro* quantification of dxs activity

The DH10B strain – araBAD promoter system was used to produce DXS at 20°C, whose catalytic activity was characterized *in vitro*. At 24h after induction, insoluble cell lysates were prepared as described above except that they were well resuspended in 100uL NPI-10 (50mM NaH_2_PO_4_, 300mM NaCl, 10mM imidazole, pH=8) instead of 2% w/v SDS. 1uL the mixture was then incubated in 20uL *in vitro* reaction solution containing 40mM Tris (pH=6), 10mM pyruvate, 20mM DL-glyceraldehyde 3-phosphate, 1mM thiamine diphosphate, 12.5 mM MgCl_2_ and 5 mM β-mercaptoethanol. The reaction was terminated by 1mL acidic extraction solution (acetonitrile/methanol/water 40:40:20, 100mM formic acid) after 2h incubation at 37°C, and formation of DXP was quantified by SPE UPLC-MS.

### SPE UPLC-MS quantification of DXP and MVAP

Concentration of DXP and MVAP in cell culture was quantified by SPE UPLC-MS. At 5h after induction, 50uL cell suspension was sampled and diluted in 1mL acidic extraction solution (acetonitrile/methanol/water 40:40:20, 100mM formic acid) and centrifuged at 16,000g for 1min. Supernatant was loaded to a cartridge holding 11 mg LC-NH2 resin (Sigma) that was activated by 200uL acidic extraction solution. The cartridge was centrifuged at 2,800g for 1min, and eluted with 100uL 1% w/v NH4OH that was subsequently neutralized by 0.75uL acetic acid. The eluate was analyzed by UPLC (Waters ACQUITY UPLC) – MS (Bruker micrOTOF II) as described below. Aqueous solution (A) containing 15 mM acetic acid and 10mM tributylamine and methanol (B) were used as mobile phase with a UPLC C18 column (Waters CSH C18 1.7μm 2.1x 50mm). The elution was done at 0.15 mL/min with gradient (start: 100% A, 1.8min: 100% A, 3.1min: 60% A, 4.9min: 60% A, 5.4min: 10% A, 9.5min: 10% A, 10min: 100% A). Electrospray ionization was used and (TOF)MS was operated to scan 50–800 m/z in negative mode with -500V end plate voltage and 4500V capillary voltage. Nebulizer gas was provided in 1bar, drying gas temperature was 9mL/min, and dry gas temperature was 200°C. Sample injection volume was 5μL. A range of m/z was extracted from the acquired data for DXP (213.0170±0.03, eluted at 5.6min) or MVAP (227.0315±0.03, eluted at 6.7min). The integrated area of signal peak at its retention time then was calculated for the metabolites with the software provided by the manufacturer. Based on the integrated area of signal, concentration of DXP and MVAP were determined by interpolating from a standard dilution of the intermediates prepared in biological matrix.

## Competing interests

The authors declare that they have no competing interests.

## Authors’ contributions

KZ and HPT conceived the study. KZ and RYZ carried out the experiments. GS and HPT supervised the study. KZ and HPT wrote the manuscript. All authors read and approved the final manuscript.

## Supplementary Material

Additional file 1 Validation of the protein solubility quantification method. Click here for file

Additional file 2 Effects of sorbitol on solubility of DXR, ISPD, ISPE, ISPF, ISPG, ISPH, IDI and ISPA. Click here for file

Additional file 3 **Expression of nonfunctional DXS R398A in *E. coli *DH10B.** Click here for file

Additional file 4 Effects of sorbitol addition on production of MEP and MEC. Click here for file

Additional file 5 Effects of fusion partners on DXS solubility. Click here for file

Additional file 6 Attempts at improving solubility of DXS by protein mutagenesis. Click here for file

Additional file 7 Addition of 500mM sorbitol did not improve lycopene yield. Click here for file

Additional file 8 Bacteria strains, plasmids and primers used in this study. Click here for file
