# Supplementary material for: Enhancing solubility of deoxyxylulose phosphate pathway enzymes for microbial isoprenoid production
Source: Microb Cell Fact. 2012 Nov 14;11:148. doi: 10.1186/1475-2859-11-148 (PMC3545872; doi:10.1186/1475-2859-11-148)
Supplement: Additional file 1 — Validation of the protein solubility quantification method. [file 1475-2859-11-148-S1.ppt]

## Slide 1
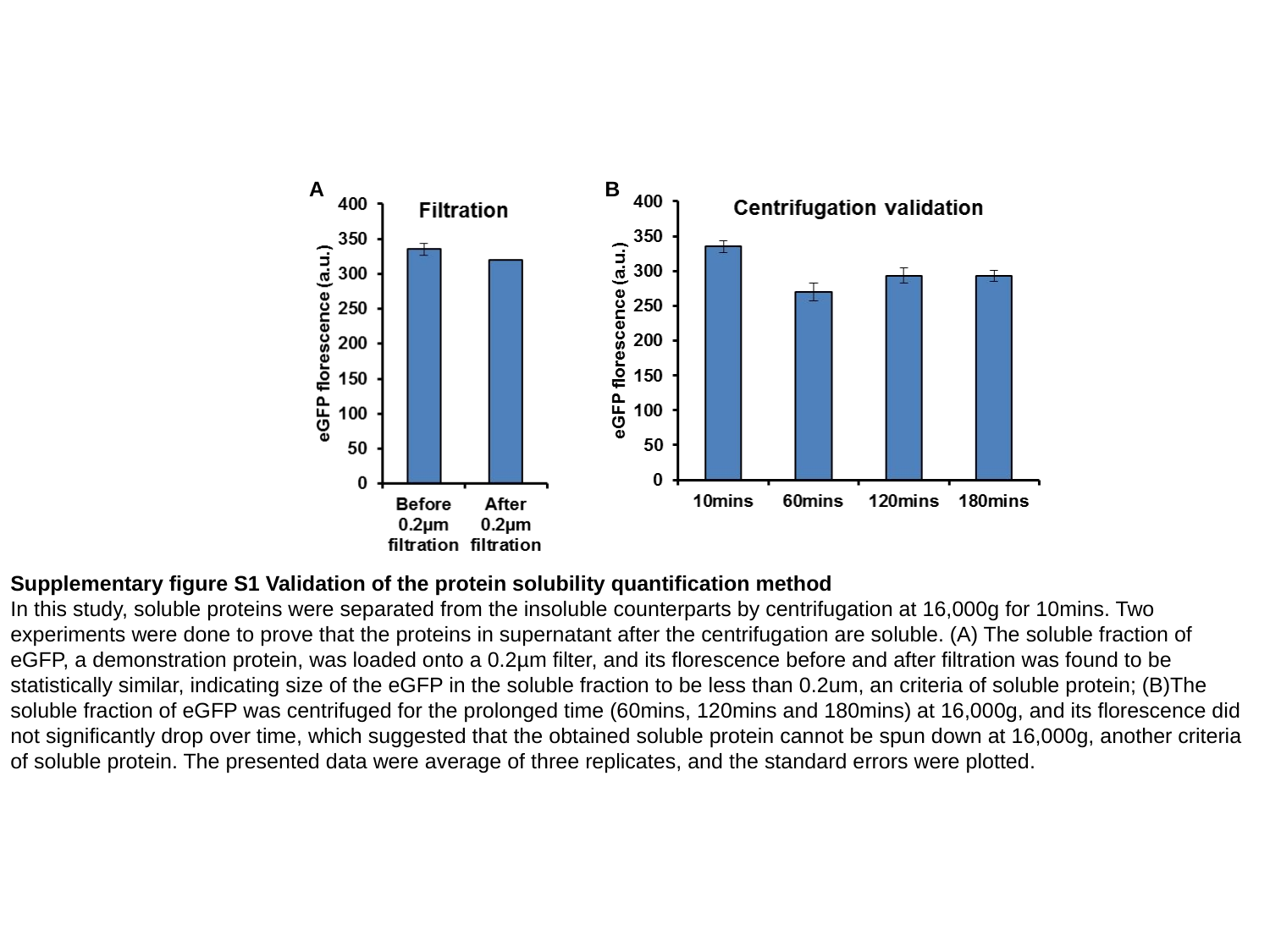

A
B
Supplementary figure S1 Validation of the protein solubility quantification method
In this study, soluble proteins were separated from the insoluble counterparts by centrifugation at 16,000g for 10mins. Two experiments were done to prove that the proteins in supernatant after the centrifugation are soluble. (A) The soluble fraction of eGFP, a demonstration protein, was loaded onto a 0.2µm filter, and its florescence before and after filtration was found to be statistically similar, indicating size of the eGFP in the soluble fraction to be less than 0.2um, an criteria of soluble protein; (B)The soluble fraction of eGFP was centrifuged for the prolonged time (60mins, 120mins and 180mins) at 16,000g, and its florescence did not significantly drop over time, which suggested that the obtained soluble protein cannot be spun down at 16,000g, another criteria of soluble protein. The presented data were average of three replicates, and the standard errors were plotted.
