# Supplementary material for: Enhancing solubility of deoxyxylulose phosphate pathway enzymes for microbial isoprenoid production
Source: Microb Cell Fact. 2012 Nov 14;11:148. doi: 10.1186/1475-2859-11-148 (PMC3545872; doi:10.1186/1475-2859-11-148)
Supplement: Additional file 2 — Effects of sorbitol on solubility of DXR, ISPD, ISPE, ISPF, ISPG, ISPH, IDI and ISPA. [file 1475-2859-11-148-S2.ppt]

## Slide 1
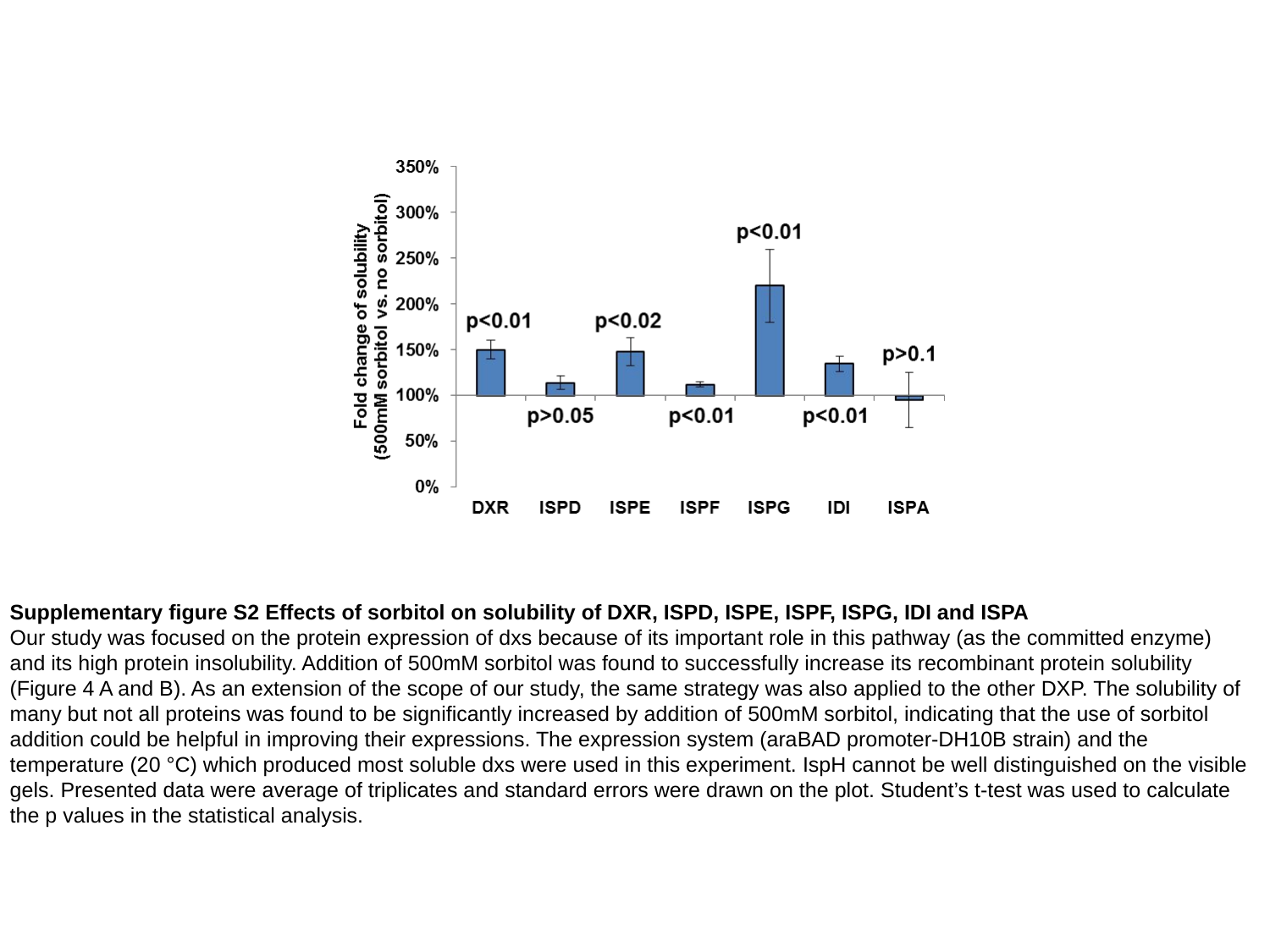

Supplementary figure S2 Effects of sorbitol on solubility of DXR, ISPD, ISPE, ISPF, ISPG, IDI and ISPA
Our study was focused on the protein expression of dxs because of its important role in this pathway (as the committed enzyme) and its high protein insolubility. Addition of 500mM sorbitol was found to successfully increase its recombinant protein solubility (Figure 4 A and B). As an extension of the scope of our study, the same strategy was also applied to the other DXP. The solubility of many but not all proteins was found to be significantly increased by addition of 500mM sorbitol, indicating that the use of sorbitol addition could be helpful in improving their expressions. The expression system (araBAD promoter-DH10B strain) and the temperature (20 °C) which produced most soluble dxs were used in this experiment. IspH cannot be well distinguished on the visible gels. Presented data were average of triplicates and standard errors were drawn on the plot. Student’s t-test was used to calculate the p values in the statistical analysis.
