# Supplementary material for: Enhancing solubility of deoxyxylulose phosphate pathway enzymes for microbial isoprenoid production
Source: Microb Cell Fact. 2012 Nov 14;11:148. doi: 10.1186/1475-2859-11-148 (PMC3545872; doi:10.1186/1475-2859-11-148)
Supplement: Additional file 3 — Expression of nonfunctional DXS R398A in E. coli DH10B. [file 1475-2859-11-148-S3.ppt]

## Slide 1
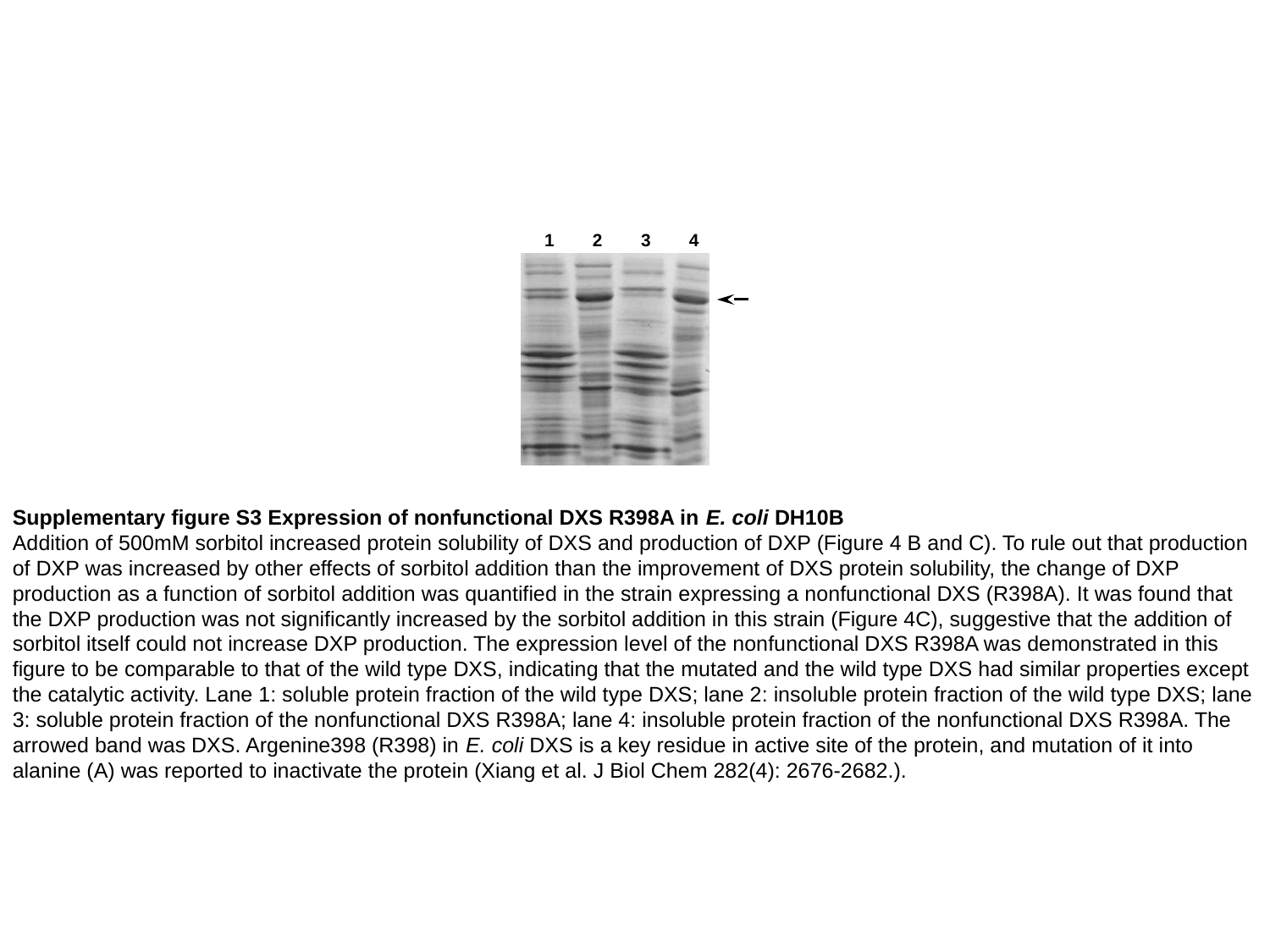

1
2
3
4
Supplementary figure S3 Expression of nonfunctional DXS R398A in E. coli DH10B
Addition of 500mM sorbitol increased protein solubility of DXS and production of DXP (Figure 4 B and C). To rule out that production of DXP was increased by other effects of sorbitol addition than the improvement of DXS protein solubility, the change of DXP production as a function of sorbitol addition was quantified in the strain expressing a nonfunctional DXS (R398A). It was found that the DXP production was not significantly increased by the sorbitol addition in this strain (Figure 4C), suggestive that the addition of sorbitol itself could not increase DXP production. The expression level of the nonfunctional DXS R398A was demonstrated in this figure to be comparable to that of the wild type DXS, indicating that the mutated and the wild type DXS had similar properties except the catalytic activity. Lane 1: soluble protein fraction of the wild type DXS; lane 2: insoluble protein fraction of the wild type DXS; lane 3: soluble protein fraction of the nonfunctional DXS R398A; lane 4: insoluble protein fraction of the nonfunctional DXS R398A. The arrowed band was DXS. Argenine398 (R398) in E. coli DXS is a key residue in active site of the protein, and mutation of it into alanine (A) was reported to inactivate the protein (Xiang et al. J Biol Chem 282(4): 2676-2682.).
