# Supplementary material for: Enhancing solubility of deoxyxylulose phosphate pathway enzymes for microbial isoprenoid production
Source: Microb Cell Fact. 2012 Nov 14;11:148. doi: 10.1186/1475-2859-11-148 (PMC3545872; doi:10.1186/1475-2859-11-148)
Supplement: Additional file 4 — Effects of sorbitol addition on production of MEP and MEC. [file 1475-2859-11-148-S4.ppt]

## Slide 1
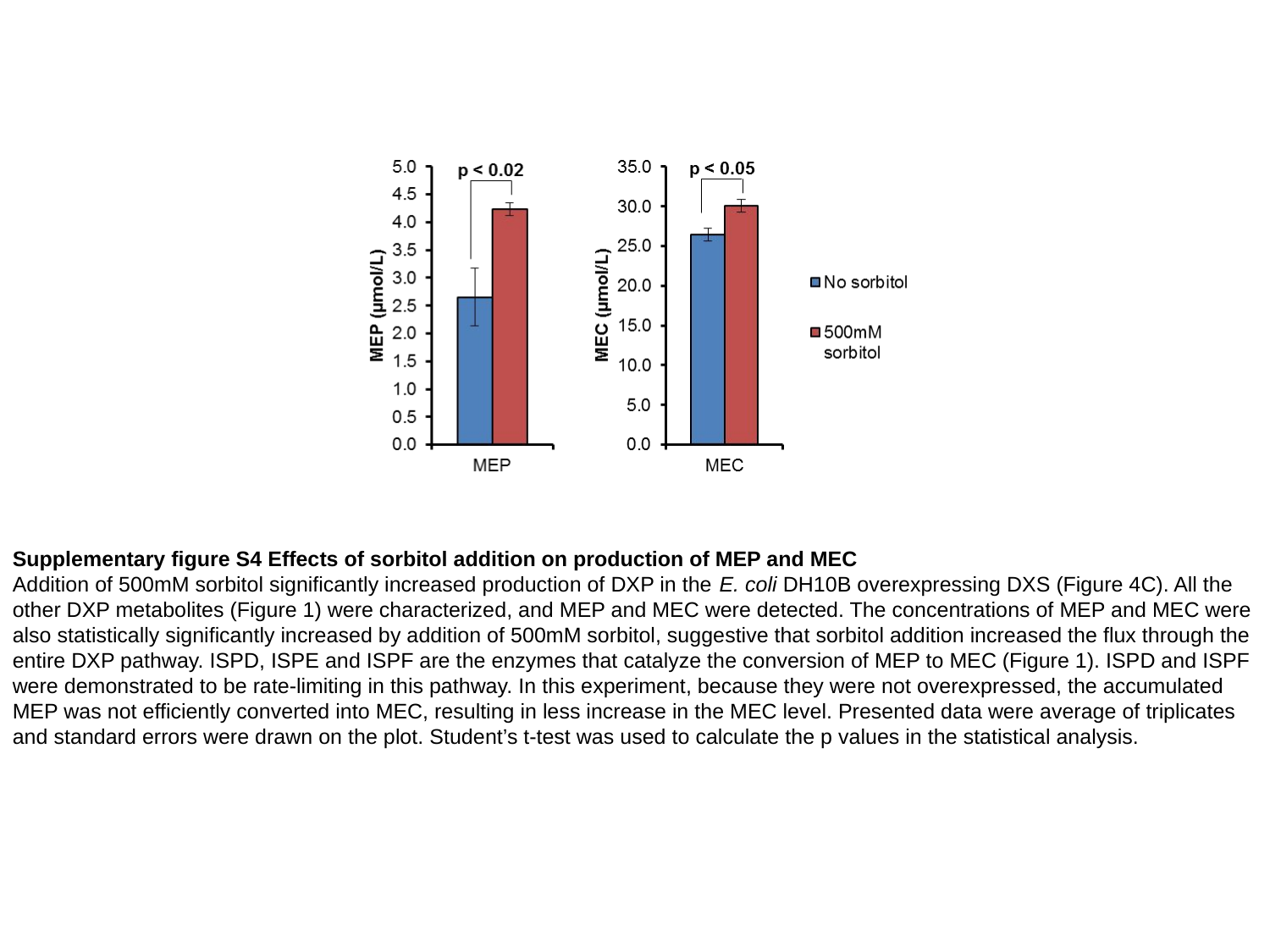

Supplementary figure S4 Effects of sorbitol addition on production of MEP and MEC
Addition of 500mM sorbitol significantly increased production of DXP in the E. coli DH10B overexpressing DXS (Figure 4C). All the other DXP metabolites (Figure 1) were characterized, and MEP and MEC were detected. The concentrations of MEP and MEC were also statistically significantly increased by addition of 500mM sorbitol, suggestive that sorbitol addition increased the flux through the entire DXP pathway. ISPD, ISPE and ISPF are the enzymes that catalyze the conversion of MEP to MEC (Figure 1). ISPD and ISPF were demonstrated to be rate-limiting in this pathway. In this experiment, because they were not overexpressed, the accumulated MEP was not efficiently converted into MEC, resulting in less increase in the MEC level. Presented data were average of triplicates and standard errors were drawn on the plot. Student’s t-test was used to calculate the p values in the statistical analysis.
