# Supplementary material for: Enhancing solubility of deoxyxylulose phosphate pathway enzymes for microbial isoprenoid production
Source: Microb Cell Fact. 2012 Nov 14;11:148. doi: 10.1186/1475-2859-11-148 (PMC3545872; doi:10.1186/1475-2859-11-148)
Supplement: Additional file 5 — Effects of fusion partners on DXS solubility. [file 1475-2859-11-148-S5.ppt]

## Slide 1
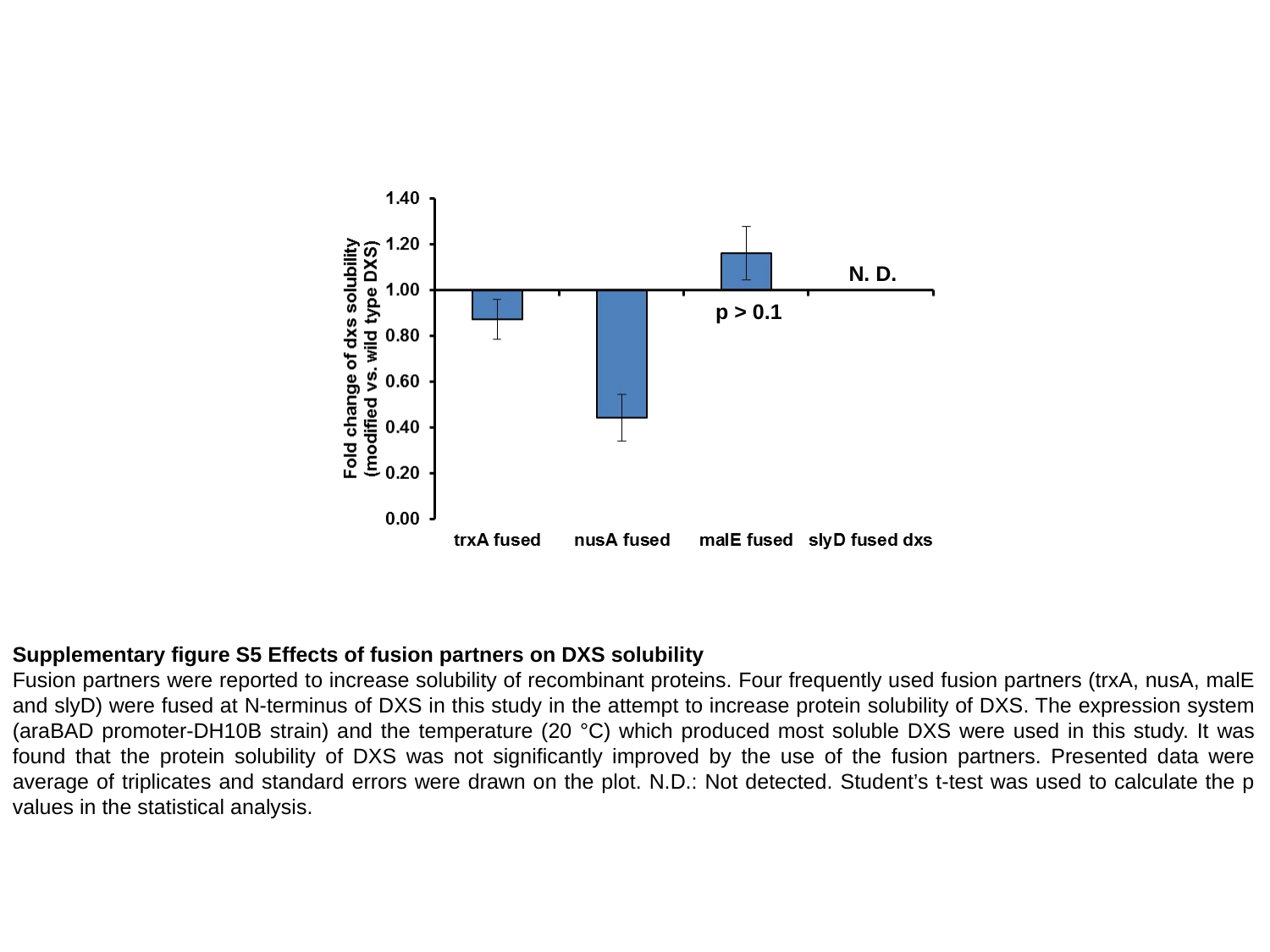

N. D.
p > 0.1
Supplementary figure S5 Effects of fusion partners on DXS solubility
Fusion partners were reported to increase solubility of recombinant proteins. Four frequently used fusion partners (trxA, nusA, malE and slyD) were fused at N-terminus of DXS in this study in the attempt to increase protein solubility of DXS. The expression system (araBAD promoter-DH10B strain) and the temperature (20 °C) which produced most soluble DXS were used in this study. It was found that the protein solubility of DXS was not significantly improved by the use of the fusion partners. Presented data were average of triplicates and standard errors were drawn on the plot. N.D.: Not detected. Student’s t-test was used to calculate the p values in the statistical analysis.
