# Supplementary material for: Enhancing solubility of deoxyxylulose phosphate pathway enzymes for microbial isoprenoid production
Source: Microb Cell Fact. 2012 Nov 14;11:148. doi: 10.1186/1475-2859-11-148 (PMC3545872; doi:10.1186/1475-2859-11-148)
Supplement: Additional file 6 — Attempts at improving solubility of DXS by protein mutagenesis. [file 1475-2859-11-148-S6.ppt]

## Slide 1
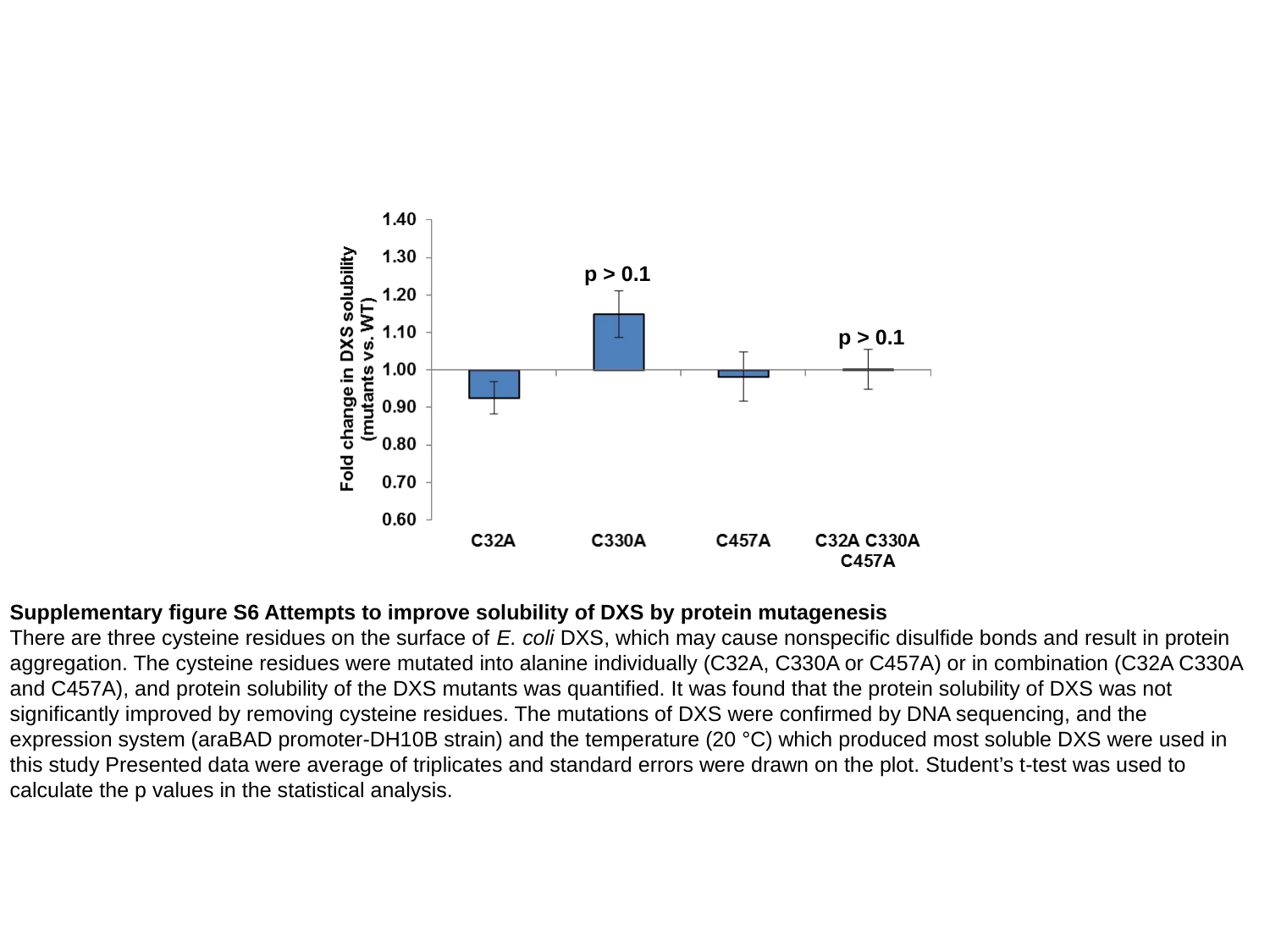

p > 0.1
p > 0.1
Supplementary figure S6 Attempts to improve solubility of DXS by protein mutagenesis
There are three cysteine residues on the surface of E. coli DXS, which may cause nonspecific disulfide bonds and result in protein aggregation. The cysteine residues were mutated into alanine individually (C32A, C330A or C457A) or in combination (C32A C330A and C457A), and protein solubility of the DXS mutants was quantified. It was found that the protein solubility of DXS was not significantly improved by removing cysteine residues. The mutations of DXS were confirmed by DNA sequencing, and the expression system (araBAD promoter-DH10B strain) and the temperature (20 °C) which produced most soluble DXS were used in this study Presented data were average of triplicates and standard errors were drawn on the plot. Student’s t-test was used to calculate the p values in the statistical analysis.
