# Supplementary material for: Enhancing solubility of deoxyxylulose phosphate pathway enzymes for microbial isoprenoid production
Source: Microb Cell Fact. 2012 Nov 14;11:148. doi: 10.1186/1475-2859-11-148 (PMC3545872; doi:10.1186/1475-2859-11-148)
Supplement: Additional file 7 — Addition of 500mM sorbitol did not improve lycopene yield. [file 1475-2859-11-148-S7.ppt]

## Slide 1
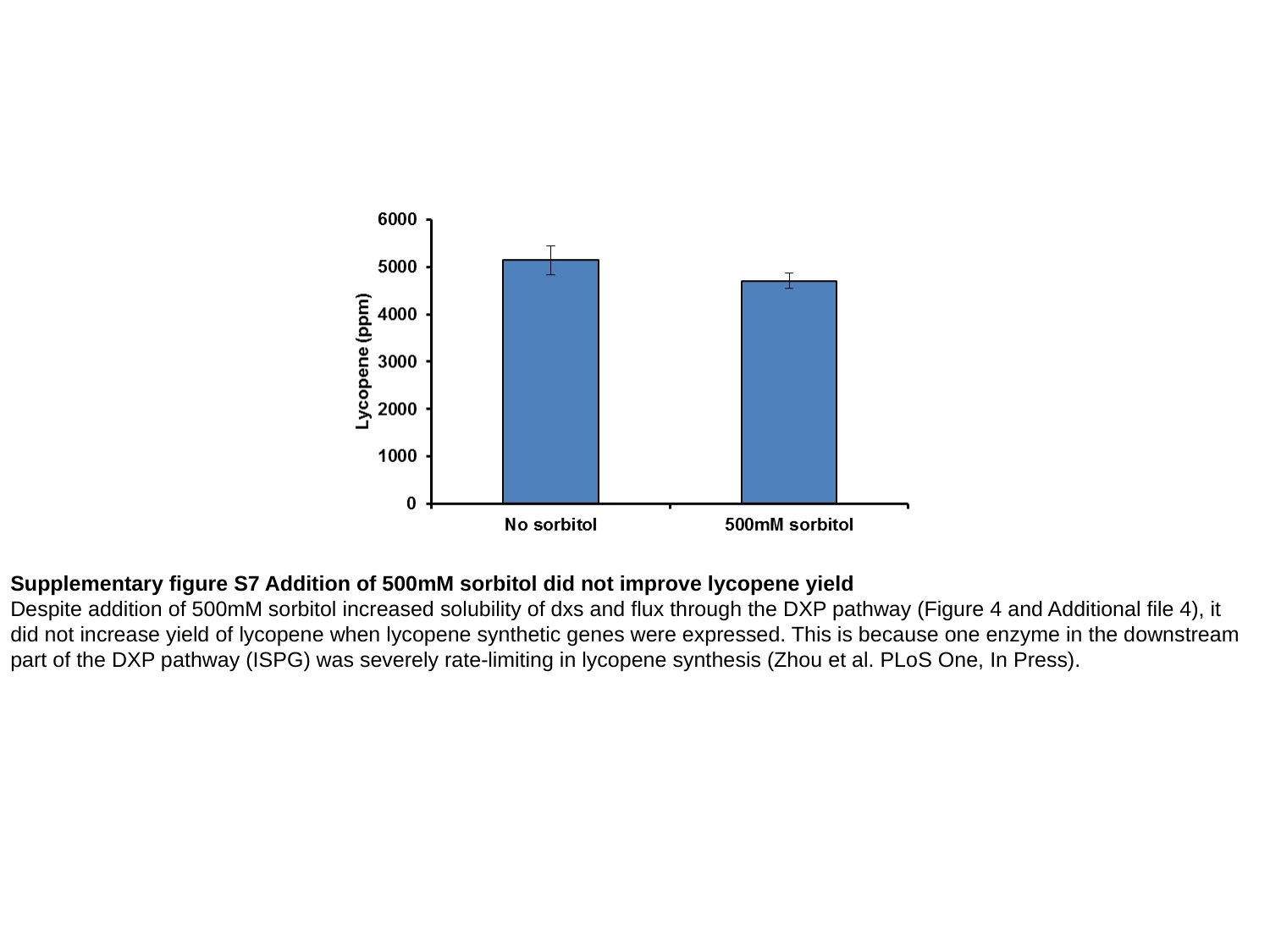

Supplementary figure S7 Addition of 500mM sorbitol did not improve lycopene yield
Despite addition of 500mM sorbitol increased solubility of dxs and flux through the DXP pathway (Figure 4 and Additional file 4), it did not increase yield of lycopene when lycopene synthetic genes were expressed. This is because one enzyme in the downstream part of the DXP pathway (ISPG) was severely rate-limiting in lycopene synthesis (Zhou et al. PLoS One, In Press).
