# Supplementary material for: Enhancing solubility of deoxyxylulose phosphate pathway enzymes for microbial isoprenoid production
Source: Microb Cell Fact. 2012 Nov 14;11:148. doi: 10.1186/1475-2859-11-148 (PMC3545872; doi:10.1186/1475-2859-11-148)
Supplement: Additional file 8 — Bacteria strains, plasmids and primers used in this study. [file 1475-2859-11-148-S8.doc]

Supplementary table S1 Bacteria strains and plasmids used in this study

| Name | Description | Reference |
| --- | --- | --- |
| *E. coli* BL21-Gold (DE3) | F^–^ ompT hsdS (r_B_^–^ m_B_^–^) dcm^+^ Tet^r^ gal λ(DE3) endA Hte | Stratagene |
| *E. Coli* DH10B | araD139 Δ(ara-leu)7697 fhuA lacX74 galK (Φ80 Δ(lacZ)M15) mcrA galU recA1 endA1 nupG rpsL Δ(mrr-hsdRMS-mcrBC) | NEB |
| *E. Coli* M15 | Lac, ara, gal, mtl, recA^+^, uvr^+^, [pREP4, lacI, kan^r^] | Qiagen |
| pACLYC | Plasmid for production of lycopene |  |
| pET-dxs | Plasmid for overexpression of dxs in *E. Coli* BL21-Gold (DE3) | This study |
| pET-dxr | Plasmid for overexpression of dxr in *E. Coli* BL21-Gold (DE3) | This study |
| pET-ispD | Plasmid for overexpression of ispD in *E. Coli* BL21-Gold (DE3) | This study |
| pET-ispE | Plasmid for overexpression of ispE in *E. Coli* BL21-Gold (DE3) | This study |
| pET-ispF | Plasmid for overexpression of ispF in *E. Coli* BL21-Gold (DE3) | This study |
| pET-ispG | Plasmid for overexpression of ispG in *E. Coli* BL21-Gold (DE3) | This study |
| pET-ispH | Plasmid for overexpression of ispH in *E. Coli* BL21-Gold (DE3) | This study |
| pET-idi | Plasmid for overexpression of idi in *E. Coli* BL21-Gold (DE3) | This study |
| pET-ispA | Plasmid for overexpression of ispA in *E. Coli* BL21-Gold (DE3) | This study |
| pBAD-dxs | Plasmid for overexpression of dxs in *E. Coli* DH10B | This study |
| pBAD-dxr | Plasmid for overexpression of dxr in *E. Coli* DH10B | This study |
| pBAD-ispD | Plasmid for overexpression of ispD in *E. Coli* DH10B | This study |
| pBAD-ispE | Plasmid for overexpression of ispE in *E. Coli* DH10B | This study |
| pBAD-ispF | Plasmid for overexpression of ispF in *E. Coli* DH10B | This study |
| pBAD-ispG | Plasmid for overexpression of ispG in *E. Coli* DH10B | This study |
| pBAD-ispH | Plasmid for overexpression of ispH in *E. Coli* DH10B | This study |
| pBAD-idi | Plasmid for overexpression of idi in *E. Coli* DH10B | This study |
| pBAD-ispA | Plasmid for overexpression of ispA in *E. Coli* DH10B | This study |
| pQE-dxs | Plasmid for overexpression of dxs in *E. Coli* M15 | This study |
| pQE-dxr | Plasmid for overexpression of dxr in *E. Coli* M15 | This study |
| pQE-ispD | Plasmid for overexpression of ispD in *E. Coli* M15 | This study |
| pQE-ispE | Plasmid for overexpression of ispE in *E. Coli* M15 | This study |
| pQE-ispF | Plasmid for overexpression of ispF in *E. Coli* M15 | This study |
| pQE-ispG | Plasmid for overexpression of ispG in *E. Coli* M15 | This study |
| pQE-ispH | Plasmid for overexpression of ispH in *E. Coli* M15 | This study |
| pQE-idi | Plasmid for overexpression of idi in *E. Coli* M15 | This study |
| pQE-ispA | Plasmid for overexpression of ispA in *E. Coli* M15 | This study |
| pBAD-malE-dxs | Plasmid for overexpression of malE fused dxs in *E. Coli* DH10B | This study |
| pBAD-trxA-dxs | Plasmid for overexpression of trxA fused dxs in *E. Coli* DH10B | This study |
| pBAD-nusA-dxs | Plasmid for overexpression of nusA fused dxs in *E. Coli* DH10B | This study |
| pBAD-slyD-dxs | Plasmid for overexpression of slyD fused dxs in *E. Coli* DH10B | This study |
| pBAD-erg12 | Plasmid for overexpression of erg12 in *E. coli* DH10B | This study |
| pBAD-dxs R398A | Plasmid for overexpression of dxs R398A | This study |
| pBAD-dxs C32A | Plasmid for overexpression of dxs C32A | This study |
| pBAD-dxs C330A | Plasmid for overexpression of dxs C330A | This study |
| pBAD-dxs C457A | Plasmid for overexpression of dxs C457A | This study |
| pBAD-dxs C32A-C330A-C457A | Plasmid for overexpression of dxs C32A-C330A-C457A | This study |

Supplementary table S2 Primers used in this study

| Name | Sequence |
| --- | --- |
| SacI-Ec_ispA(No ATG) | GCTTAGAGCTCGACTTTCCGCAGCAACT |
| Ec_ispA-XhoI | GTAACCTCGAGTTATTTATTACGCTGGATGA |
| SacI-Ec_ispD(No ATG) | GCTTAGAGCTCGCAACCACTCATTTGGA |
| Ec_ispD-XhoI | GTAACCTCGAGTTATGTATTCTCCTGATGGATG |
| SacI-Ec_ispE(No ATG) | GCTTAGAGCTCCGGACACAGTGGCCCTC |
| Ec_ispE-XhoI | GTAACCTCGAGTTAAAGCATGGCTCTGTGC |
| SacI-Ec_ispF(No ATG) | GCTTAGAGCTCCGAATTGGACACGGTTT |
| Ec_ispF-XhoI | GTAACCTCGAGTCATTTTGTTGCCTTAATGA |
| SacI-Ec_ispG(No ATG) | GCTTAGAGCTCCATAACCAGGCTCCAAT |
| Ec_ispG-XhoI | GTAACCTCGAGTTATTTTTCAACCTGCTGAAC |
| SacI-Ec_ispH(No ATG) | GCTTAGAGCTCCAGATCCTGTTGGCCAA |
| Ec_ispH-XhoI | GTAACCTCGAGTTAATCGACTTCACGAATATC |
| SacI-Ec_dxr(No ATG) | GCTTAGAGCTCAAGCAACTCACCATTCTGGG |
| Ec_dxr-XhoI | GTAACCTCGAGTCAGCTTGCGAGACGC |
| SacI-Ec_dxs(No ATG) | GCTTAGAGCTCAGTTTTGATATTGCCAAATA |
| Ec_dxs-XhoI | GTAACCTCGAGTTATGCCAGCCAGGC |
| SacI-Ec_idi(No ATG) | GCTTAGAGCTCCAAACGGAACACGTCAT |
| Ec_idi-XhoI | GTAACCTCGAGTTATTTAAGCTGGGTAAATGC |
| NcoI-trxA | GCCCATGGGTAGCGATAAAATTATTCACCTGAC |
| trxA-SacI | GCGAGCTCCGCCAGGTTAGCGTCGA |
| NcoI-slyD | GCCCATGGGTAAAGTAGCAAAAGACCTGGTGG |
| slyD-SacI | GCGAGCTCGTGGCAACCGCAACCG |
| NcoI-malE | GCCCATGGGTAAAATCGAAGAAGGTAAACTG |
| malE-SacI | GTAACGAGCTCAGTCTGCGCGTCTTTCA |
| NcoI-nusA | GCCCATGGGTAACAAAGAAATTTTGGCTGTAG |
| nusA-SacI | GCGAGCTCCGCTTCGTCACCGAACC |
| SacI-Sc.ERG12-f | GCGAGCTCTCATTACCGTTCTTAACTTCTGC |
| Sc.ERG12-XhoI-r | GCCTCGAGTTATGAAGTCCATGGTAAATTCG |
| dxs (CG1192GC)-F | CTTTCCTGCAAGCCGCCTATGATCAGGT |
| Dxs TG94GC R | CGGCGCAGTTCGTCGGCGAGTTTCGGTAAACTCTCTTTCG |
| Dxs TG988GC R | TTCGCTGCCGTTTCGGCCAACCAGTCGCCAAAGA |
| Dxs TG1369GC F | GCGATGAAAACGAAGCTCGCCAGATGCTCTATACCG |

Underlined text: restriction enzyme site
